# GC-MS profiling of *Pistachio vera* L., and effect of antioxidant and antimicrobial compounds of it's essential oil compared to chemical counterparts

**DOI:** 10.1038/s41598-023-48844-5

**Published:** 2023-12-07

**Authors:** Fatemeh Shahdadi, Sepideh Khorasani, Ali Salehi-Sardoei, Farshad Fallahnajmabadi, Bahman Fazeli-Nasab, R. Z. Sayyed

**Affiliations:** 1 Food Science and Technology Department, Faculty of Agriculture, University of Jiroft, Jiroft, 7867155311 Iran; 2 Food Science and Technology Department, Faculty of Agriculture, Shahid Bahonar University of Kerman, Kerman, 7616913439 Iran; 3 Department of Horticulture, Faculty of Plant Production, Gorgan University of Agriculture and Natural Resources, Gorgan, Iran; 4 Chief Executive of Pestaco Corporation, Tehran, 1417935840 Iran; 5 Department of Agronomy and Plant Breeding, Agriculture Institute, Research Institute of Zabol, Zabol, 9861335884 Iran; 6 Department of Microbiology, PSGVP Mandal’s S I Patil Arts, G B Patel Science and STKVS Commerce College, Shahada, 425409 India

**Keywords:** Microbiology, Plant sciences

## Abstract

All elements of the pistachio tree are considered raw pistachio by-products. The soft hull makes up the majority of these by-products. It contains proteins, fats, minerals, vitamins, phenolics contents (TPC), and antioxidants. Early smiling pistachios are one of the most important sources of pistachio contamination with aflatoxin in the garden and processing stages. The present study aimed to evaluate pistachio hull essential oil (EO) composition, and antioxidant and antimicrobial properties under in vitro conditions. TPC, antioxidant, and antimicrobial activity were measured using the Folin–Ciocalteu reagent, 2,2-diphenyl-1-picrylhydrazyl (DPPH) free radical scavenging method, and serial dilution titration method, respectively. A gas chromatography system with a mass spectrometer (GC-MS) was utilized to determine the chemical components of the EO. The findings revealed that the quantity of TPC and anti-radical activity in IC_50_ were 245.43 mg gallic acid/mL and 206.32 µL/L, respectively. The free radical absorption activity of DPPH (%) increased with EO content. The inhibitory activity of EO on *Staphylococcus aureus* and *Bacillus subtilis* was much lower than that of streptomycin and penicillin. *Aspergillus flavus* was effectively inhibited by pistachio hull EO, comparable to fluconazole. The results obtained from GC-MS showed that the major compounds in pistachio hull essential oil include α-pinene (47.36%), terpinolene (10.57%), limonene (9.13%), and L-bornyl acetate (8.57%). The findings indicated that pistachio hull EO has potent antibacterial and antioxidant components and can be employed as a natural antimicrobial and antioxidant in food systems.

## Introduction

During the processing of agricultural products, a significant amount of by-products are produced, many of which are usually unused, discarded, and considered as one of the causes of environmental pollution^1–6^. One of Iran's most significant agricultural products is the pistachio (*Pistacia vera*), grown in numerous regions on an extensive scale. Iran is the largest producer of pistachios in the world, with an annual production of 478,600 tons per year^7^ and in Iran, according to the statistics of the Ministry of Agriculture- Jahad, in 2016, Kerman, Yazd, Razavi Khorasan, Fars, and South Khorasan provinces, respectively, have the most amount of pistachio production^8^. The main by-products of pistachios are mainly soft green hulls, clusters, leaves, and small amounts of kernels and wood husks^9^.

Fresh pistachios have about 40% soft hull, removed by machines after harvesting, which is usually not used much except as animal feed. Research has shown that the pistachio green hull includes dry matter: 32.64%; crude protein: 11.24%; crude fiber: 15.38%; ash: 12.3%; crude fat: 5.79%, essential fatty acids and various minerals^10,11^. This product is reported to contain hydrolyzable tannins that inhibit the production of aflatoxins by fungi^12^. The findings demonstrate that soybean oil was effectively kept from oxidizing at 60 °C by pistachio green hull extract at a concentration of 0.02–0.06%^13^.

Ayatollahi et al.^14^ have determined the chemical composition of hull EO of two pistachio samples from Shiraz city and reported that the main components of EO from sample 1 were limonene (25.9%) and terpinolene (24.13%). Similarly, the main components of essential oil from sample 2 were limonene (47.69%) and terpinolene (24.08%).Forty-three compounds and fifteen compounds were detected in sample 1 and sample 2 essential oils. Monoterpene hydrocarbons with high percentages of limonene (25.9%) and terpinolene (24.13%) had the highest amount in sample 1 essential oil (57.65%). These compounds were more predominant (94.86%) in sample 2 essential oil, with limonene (47.69%) and terpinolene (24.08%) as main components.

The presence of *A. flavus* on growing pistachio fruits before harvest has been proven^15^ and it has been reported that the infection is related to the exposure of pistachio kernels to airborne spores due to the splitting of the green hull^16^. It has also been determined that the infection with *A. flavus* and *A. parasiticus* in early-smiling pistachios infected with navel orange worm (*Amyelois transitella*) is higher than in early-smiling pistachios not infected with NOW, and pistachios with healthy green hull (without cracking) and closed pistachios are free of aflatoxin^17,18^.

The investigation of the contamination of early-ripening and irregularly cracked pistachios showed that pistachios with wrinkled hull were more than twice infected with *A. niger* and more than 3 times with *A. parasiticus* and *A. flavus* compared to soft pistachios with soft outer hull^19^. Pistachios infected with *A. transitella* accounted for 84% of the total aflatoxin. The green hull of the smiling pistachios had a small amount of aflatoxin^20^.

Early cracking of pistachios, which is one of the sources of contamination, occurs in pistachios that are not in a normal state, and their outer hull splits along the groove of the wooden shell, and this causes the pistachio kernel to be exposed to mold^21,22^. Due to the fact that so far no research has been done regarding the contamination of Zodkhandan pistachios with fungi, aflatoxin and pests.

It evaluated the chemical composition of pistachio green hull EO from Tunisia and reported that monoterpene hydrocarbons, which mainly contained alpha-pinene and terpinolene, were present throughout the fruit development process^23^. Due to the fact that the outer hull of pistachios makes up a large amount of fruit weight and pistachios are produced in large quantities in Iran, thus there will be a lot of soft hulls produces. The presence of antioxidant and antimicrobial compounds in the pistachio green hull has been shown in many researchs. Also, due to the fact that pistachio green hull is produced in a large amount, it is possible to extract antimicrobial and antioxidant substances from its EO. Considering that there has been no research on the effect of pistachio hull essential oil as a by-product of pistachio processing on pistachio post-harvest fungi, The aim of this study was to investigate the essential oil composition of Ripe pistachio hulls of the "Ahmad-Aghaei" cultivar based on their chemical and antimicrobial compounds and their effect on pathogenic bacteria and fungal.

## Materials and methods

### Plant collection

The collection of pistachio plant material was performed according to institutional, national, and international guidelines. All methods were carried out in accordance with relevant institutional, national, and international guidelines. It was identified by Pistachio Research Center, Iran (Herbarium number: A213-Ahmad-Aghaei (*Pistacia vera*)) and examined by Dr. Ali Reza Sirousmehr, botanist of University of Zabol, Zabol, Iran.

**Figure 1:**
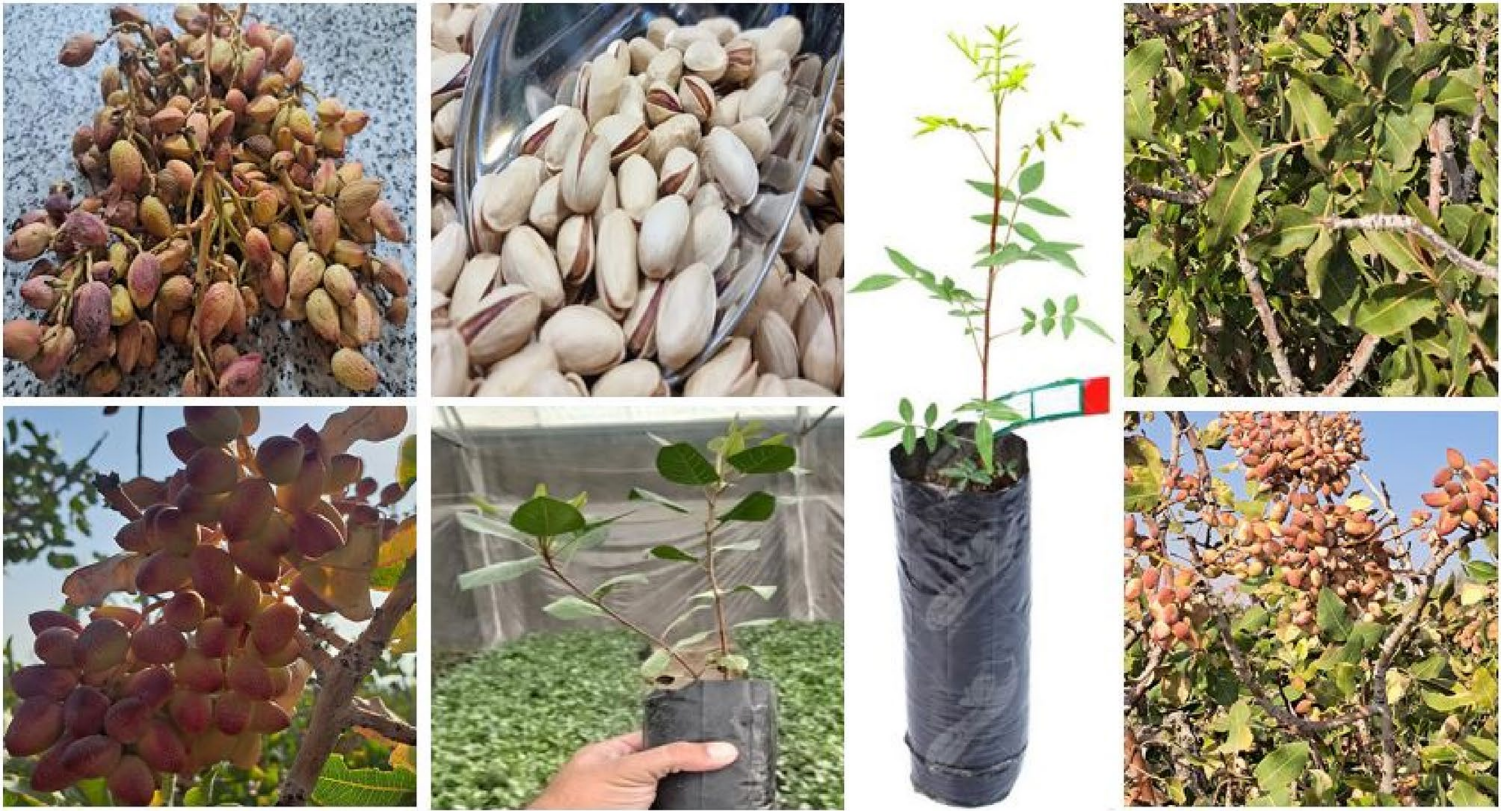
The characteristic of some tissues of *Pistacia vera*, Ahmad-Aghaei cultivar.

The primary pistachio cultivars in Iran include the commercially important variants of Jumbo (Kalleh-Ghuchi), Long (Ahmad-Aghaei) (Fig. 1), Round (O'hadi), and Super-long (Akbari). The Long (Ahmad-Aghaei) cultivar has an exceptionally high yield and excessive biennial bearing. The Long (Ahmad-Aghaei) variety of pistachios is genetically more biennial bearing. Because this cultivar is more sensitive to fungal growth than the other cultivars indicated above, prompt harvesting and quick processing of the crop are crucial^24^.

Ripe pistachio hulls of the "Ahmad-Aghaei" cultivar from a ten-year-old field were collected between September and October 2021 from Agricultural Jihad Management Organization of Rafsanjan city (Longitude 55˚ 51́ and 49̋, latitude 30˚ 25́ and 43̋, altitude 1572 m). Ahmad-Aghaei cultivar is a commercial and breaded cultivar. In all the world, the use of the modified and breeded cultivars do not require a license, because they are sold and cultivated all over the world. But in this research, the authors went to Agricultural Jihad Management Organization of Rafsanjan city and has gotten permission to use this cultivar (Ahmad-Aghaei) from the head of this department.

Rafsanjan is a city in the southeast of Iran and the northwest of Kerman province, which is located 115 km from Kerman metropolis. The city of Rafsanjan is bordered by Zarand to the northeast, Bafaq, Mehriz and Anar to the northwest, Babak to the west, Sirjan to the southwest, Bardsir to the south, and Kerman to the east. Rafsanjan is the world's largest pistachio exporter and the world's largest hand-planted forest is located in this city. Rafsanjan city has a semi-desert climate and summers are more or less hot and winters are cold. Of course, there are many cold and mountainous places around this city. The average annual rainfall of this city is 100 mm. The height of this city is 1,528 m above sea level. Of course, there are areas with a height of 2,300 m and above in the vicinity of this city. The lowest recorded temperature in Rafsanjan is -17 degrees Celsius and the highest recorded temperature is + 43 degrees Celsius. The months of "April", "November", "December", "January" and "February" have recorded the most rainfall in Rafsanjan.

The main pistachio producing provinces in Iran include Khorasan, Kerman, Yazd and Semnan. In total, 200,000 tons of pistachio products are harvested from the gardens of Iran, of which 65,000 tons are produced in Rafsanjan.

Since the species in question is widely distributed throughout the country, no certificate or permission was required for gathering the samples, however, the dean of the Vice President of the Agricultural Jihad Management Organization (Rafsanjan) was verbally consulted before sampling.

### Preparation of plant raw materials and essential oils

Ripe pistachio hulls of the "Ahmad-Aghaei" cultivar was dried in the shade using hot summer air and powdered by an electric mill.

The Clevenger system extracted the EO in the pistachio hull. First, dried pistachio hull (150 g) and distilled water (2000 mL) were poured into the balloon and placed on the heater. Then the balloon was connected to the condenser and the condenser to the burette. Finally, the heater turned on. The water valve connected to the condenser was opened. The collection was kept in this state for 4 h until all the essential oils were extracted. Then the heater turned off. The sum of water and essential oil was inside the burette because essential oils are insoluble in water; essential oil and water did not mix and formed two phases. Therefore, the essential oil was easily separated from the water. Sodium sulfate was employed to dry out the EO samples after the extraction procedure^25^.

### Determination of TPC

TPC content was determine according to the method of Bajalan et al.^21^. Briefly, 20 μL of essential oil were mixed with 300 μL of Na_2_CO_3_ solution (20%), then 1.16 mL of distilled water and 100 μL of Folin–Ciocalteu reagent (80%) added to mixture after 1 min and 8 min, respectively. Subsequently, the mixture was incubated in a shaking incubator at 40 °C for 30 min and its absorbance was measured at 760 nm. Gallic acid was used as a standard for calibration curve. The phenolic content was expressed as gallic acid equivalents by using the following linear equation obtained from calibration curve:

$${\text{A }}\left( {{76}0{\text{ nm}}} \right) = {12}.{\text{722C}} + 0.00{34},{\text{R2 }} = 0.{9994}$$

where A is the absorbance and C is the concentration as gallic acid equivalents (mg/mL)^26^.

### Determination of anti-radical activity

Different concentrations of essential oil in methanol were prepared to determine the anti-radical activity. Briefly, 0.5 mL of a 0.2 mM methanolic solution of DPPH was mixed with 3 mL of essential oil (with 50, 100, 250, 500, 800 and 1000 μg/mL concentrations). The mixture was then homogenized vigorously and left for 30 min in the dark place (at room temperature). Its absorbance was measured at 517 nm with a UV-Visible Spectrophotometer (UV 160A, Shimadzo, Japan). DPPH free radicals scavenging capacity was calculated using the following formula^27^.

$${\text{DPPH }}\left( {\text{\% }} \right) = \frac{{{\text{Control Absorbance}}{-}{\text{ Sample Absorbance}}}}{{\text{Control Absorbance}}} \times 100$$

The ability of the samples to inhibit radicals was evaluated using the IC_50_ factor. The IC_50_ value represents the concentration that effectively absorbs 50% of the free radicals. BHT (as a synthetic antioxidant) was used to compare the inhibition of DPPH free radicals^28^.

### Identification of EO chemical components

The method of performing gas chromatography was as follows: the oil was injected into a gas chromatography equipped with a mass spectrometer- (GC-MS) (Shimadzu 2010 Plus model, Japan) to identify the essential oil compounds. The system has a capillary column that is 30 m long, has an inner diameter of 250 µm, and an inner layer thickness of 0.25 µm. The oven temperature was scheduled to rise from 60 °C (0 min) to 275 °C at a rate of 5 °C/minutes, and the injector temperature was 230 °C. Helium served as the carrier gas. After injecting essential oil (1.0 μL) into the device and observing the chromatogram spectrum, using the retention time (Rt), and mass spectrum, comparing with compounds in the National Institute Standard and Technology (NIST) database, identifying essential oil compounds and determining their quantitative percentage were done.

First, 15 drops of essential oil were poured into a test tube. Then 7 cm^3^ of n-hexane and 2 cm^3^ of methanolic potassium solution (2 M) with gas chromatography grade were added. The mixture was shaken vigorously for several seconds. Then it was placed in a water bath for 15 to 30 min at a temperature of 55 ± 5˚C. After this period, 3 cm^3^ of the upper phase was removed and passed through a sieve containing dry sodium sulfate. 4 µL of the filtered sample was injected into the device. After injection, the essential oil enters the column through the mobile phase, and then due to heat, they are spread between the gas and the stationary phase, which may be dissolved or absorbed. According to the polarity of the column used, the more polar the desired substance is, the slower the sample is removed. Considering the time required to remove the object from the column and the volume of gas required to remove the object from the column, the sample was evaluated and determined by detectors^29^.

### Antibacterial and antifungal activities

#### Source of microbial cultures

A serial dilution titration approach was used to test the samples antimicrobial effectiveness to establish the minimal inhibitory concentration (MIC) and minimal bactericidal concentration (MBC). The bacteria (*Staphylococcus aureus* ATCC25923 and *B. subtilis* ATCC6051) were cultured at 37 °C in Mueller Hinton Broth (MHB) medium. The agent was diluted one-fold serially in a 100 μL, and 100 μL of bacteria were added to the microtiter plates to provide a final inoculum of 5 × 10^5 CFU^/mL.

The plates were incubated for 24 h at 37 °C. After this period, the first tube with no visible turbidity was determined as MIC. Then, 100 µL of the MIC well's 24 h inhibitory concentration test sample and subsequent concentrations were plated on Mueller Hinton Agar (MHA) media and incubated at 37 °C. The highest dilution of the essential oil in which no fungi growth was observed was the MBC. MBC/MIC ratio was used to assess antibacterial activity. When MBC/MIC was less than or equal to 4, the effect was regarded as bactericidal, but if the ratio of MBC/MIC was more than 4, the result was bacteriostatic^30^.

A serial dilution titration approach was used to examine the sample's fungal efficacy to establish the MIC and Minimum Fungicidal Concentrations (MFC). The fungi (*A. flavus* ATCC24109 and *Aspergillus parasiticus* ATCC28285) were cultured at 37 °C in the Sabouraud Dextrose broth (SDB) diluted in this medium. The micro-titer plates were filled with one-fold serial dilutions of the agent in a volume of 100 μL, then 100 μL of fungi to produce a final inoculum of 1 × 10^5^ spores. Positive control without samples and negative control with media alone were maintained for each set of experiments. The plates were incubated for 48 h at 37 °C. After this period, the tubes' turbidity and fungi growth were evaluated compared to the controls. The tubes with the lowest essential oil concentration had no fungal growth or turbidity observed, which were considered MIC. Following that, 100 µL of the 44 h inhibitory concentration test sample (MIC well) and its subsequent concentrations were plated on Sabouraud Dextrose Agar (SDA) and incubated at 25 °C. The MFC was the highest essential oil dilution in which no fungi growth was observed. MFC/MIC ratio was used to assess antifungal activity. When MFC/MIC was less than or equal to 4, the effect was regarded as fungicidal, but if the ratio of MBC/MIC was more than 4, the result was fungistatic^31^.

In addition, the MIC and MBC of several antibiotics, including streptomycin, penicillin, nystatin, and fluconazole against bacterial and fungal strains, were also determined by the serial dilution titration method. In the end, MIC and MBC results related to antibiotics and essential oil against bacterial and fungal strains were compared.

### Statistical analysis

Antioxidant and antimicrobial activity data were analyzed in a completely randomized design using SPSS: 21 software. Means were compared using Duncan's test at the 5% level.

### Ethics approval and consent to participate

No humans or animals were used in the present research.

## Results

### Chemical compounds of EO

**Figure 2:**
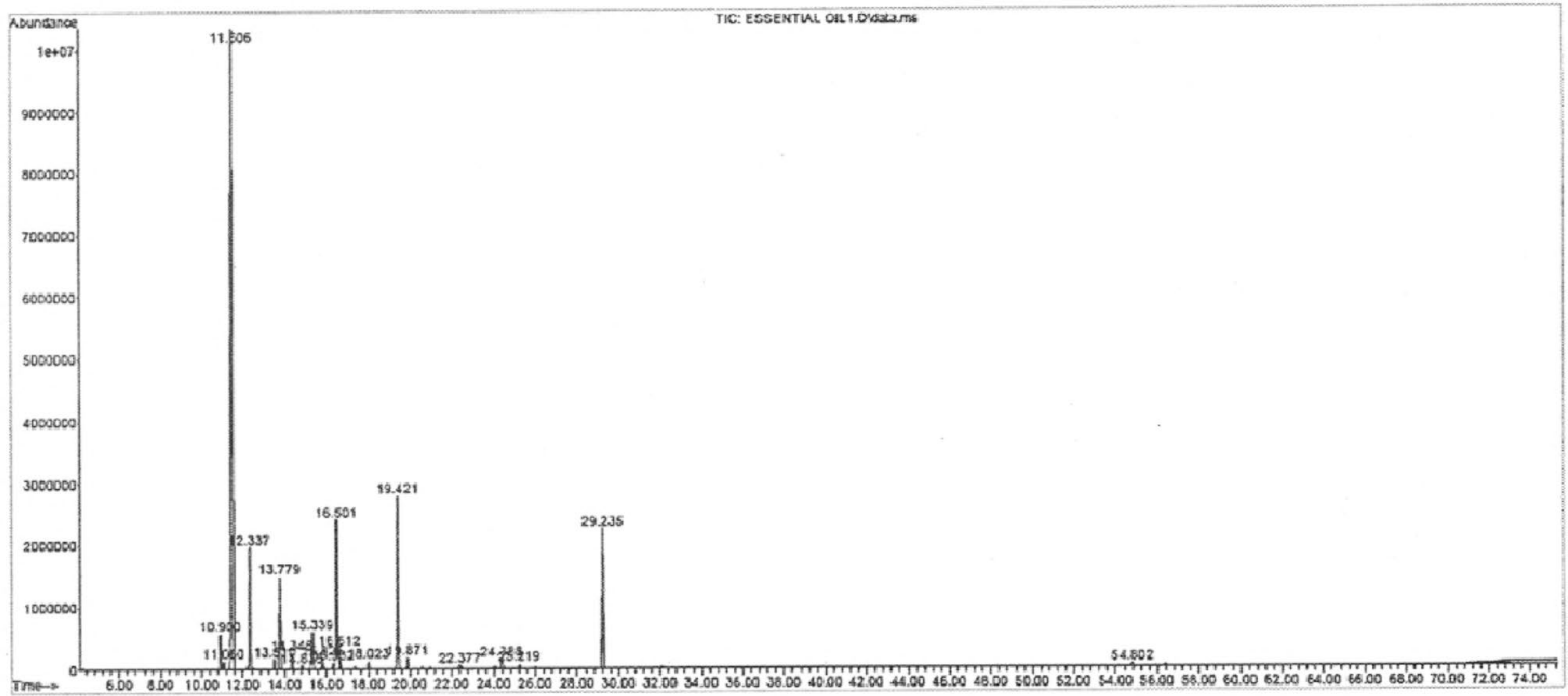
GC-MS profile of Compounds identified in pistachio soft hull essential oil.

In this study, the essential oil yield was 0.59%. Table 1 shows the compounds identified in pistachio hull essential oil by GC-MS. The pistachio hull contained a total of 19 chemicals that represented 99.54% of all substances. The main components of pistachio hull essential oil included α-pinene (47.36%), terpinolene (10.57%), limonene (9.13%), L-bornyl acetate (8.57%), Camphene (7.3%), β-Pinene (5.39%), and δ-3-Carene (2.84%) (Fig. 2).

**Table 1:** Compounds identified in pistachio soft hull essential oil.

RT	%	Components	KI	Type
10.90	2.00	Tricyclene	926	MH
11.06	0.44	α -Thujene	930	MH
11.51	47.36	α -Pinene	939	MH
12.34	7.03	Camphene	954	MH
13.51	0.56	Sabinene	975	MH
13.78	5.39	β -Pinene	979	MH
14.35	0.85	Myrcene	990	MH
14.84	0.20	δ-2-Carene	1002	MH
15.34	2.84	δ-3-Carene	1011	MH
15.83	0.65	α-Terpinene	1017	MH
16.34	0.38	ρ-Cymene	1024	MH
16.50	9.13	Limonene	1029	MH
16.62	1.29	β-Phellanderene	1030	MH
18.02	0.44	γ-Terpinene	1059	MH
19.42	10.57	Terpinolene	1088	MH
19.87	0.67	ρ-Cymenene	1091	MH
22.38	0.28	1,3,8-ρ-Menthatriene	1138	MH
24.39	0.88	*cis*-Citral	1216	MO
29.23	8.57	*L*-Bornyl acetate	1285	MO
	99.54	Total Identified		

*MH* Monoterpene hydrocarbons, *MO* Oxigenated monotepenes.

### Descriptive statistics related to the percentage of DPPH free radical scavenging from sample and BHT

The descriptive statistics related to the percentage of DPPH free radical scavenging in the sample and BHT are presented in Table 2. Based on the standard deviation values, DPPH scavenging (%) of BHT had the lowest value. The results showed that the treatments under various concentrations have different characteristics. The descriptive statistics are general information about the evaluated variables in the tested treatments and help the researchers to have a better and more accurate understanding of the investigated parameters.

**Table 2:** Descriptive statistics related to Percentage of DPPH free radical scavenging from sample and BHT*.*

Trait	Mean	Standard variation	Minimum	Maximum
DPPH scavenging (%) of sample	63.925	22.329	35.8	85.6
DPPH scavenging (%) of BHT	79.15	18.101	55.9	98.4

### TPC and antioxidant activity

**Figure 3:**
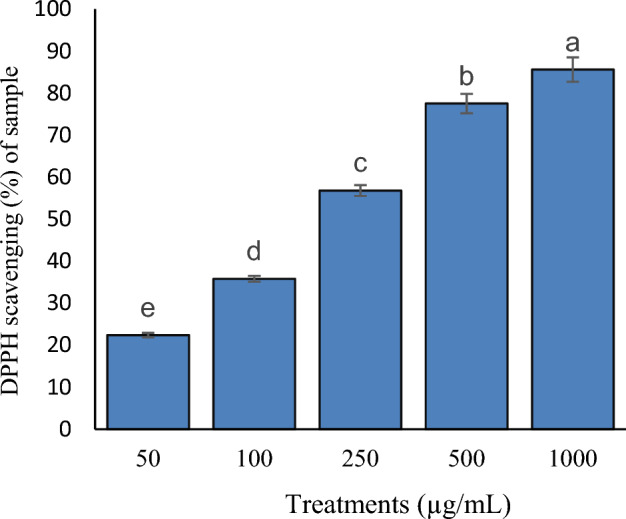
Percentage of DPPH scavenging (%) of sample from different concentrations of essential oil.

**Figure 4:**
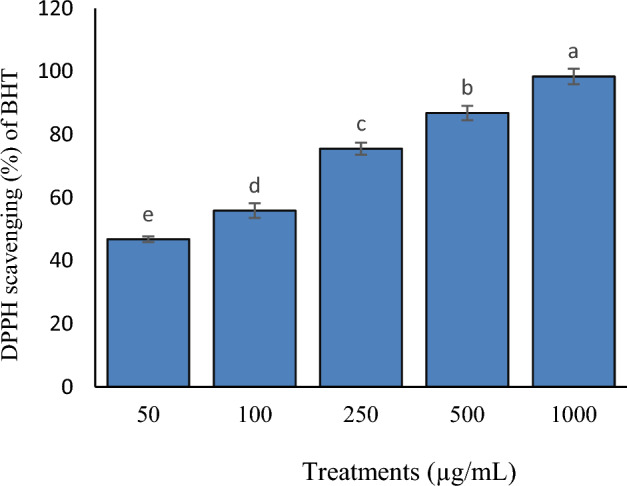
Percentage of DPPH scavenging (%) of BHT.

Table 3 shows pistachio hull essential oil's phenolics contents and IC_50_. The results of Figs. 3 and 4 show that the percentage of removal of DPPH free radicals increases with increasing concentration. At higher concentrations of EO, the amount of TPC increases. Since there are more hydroxyl groups in the reaction medium, there is a chance that free radicals could receive hydrogen as a donation, and this has an inhibitory effect.

**Table 3:** Phenolics contents and IC50 of essential oil from pistachio green hull.

Essential oil	Phenolics compounds (mg gallic acid/mL)	IC_50_ (µL/L) of sample
	245.43 ± 2.41	206.32 ± 5.54

**Figure 5:**
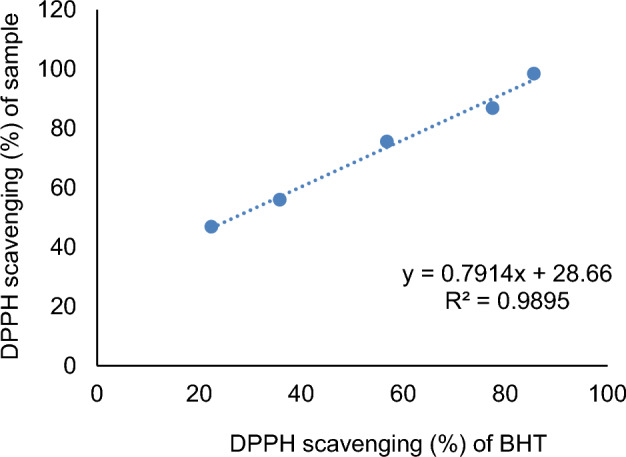
Results of DPPH scavenging (%) of sample regression linear against DPPH scavenging (%) of BHT.

The changes of DPPH scavenging (%) of sample against DPPH scavenging (%) of BHT. According to the regression coefficient, a change of one unit in DPPH scavenging (%) of BHT (µg/mL) led to a change of 0.7914 units in DPPH scavenging (%) of sample (Fig. 5).

### Antibacterial activity of EO

Descriptive statistics related to MIC and MBC of Essential oil, Streptomycin, Penicillin on *S. aureus* are presented in Table 4. Based on standard deviation, both MIC and MBC had the same values.

**Table 4:** Descriptive statistics related to The MIC of essential oil, Streptomycin (Just for Comparison), Penicillin (Just for Comparison) on *S. aureus.*

	Mean	Standard variation	Minimum	Maximum
MIC (µg/mL)	177.4	21.411	0.97	500
MBC (µg/mL)	177.4	21.411	0.97	500

Descriptive statistics related to MIC and MBC of Essential oil, Streptomycin, Penicillin on *B. subtilis* are presented in Table 5. Based on standard deviation, both MIC and MBC had the same values.

**Table 5:** Descriptive statistics related to The MIC of Essential oil, Streptomycin (Just for Comparison), Penicillin (Just for Comparison) on *B. subtilis.*

	Mean	Standard variation	Minimum	Maximum
MIC (µg/mL)	89.84	8.29	3.9	250
MBC (µg/mL)	89.84	8.29	3.9	500

**Figure 6:**
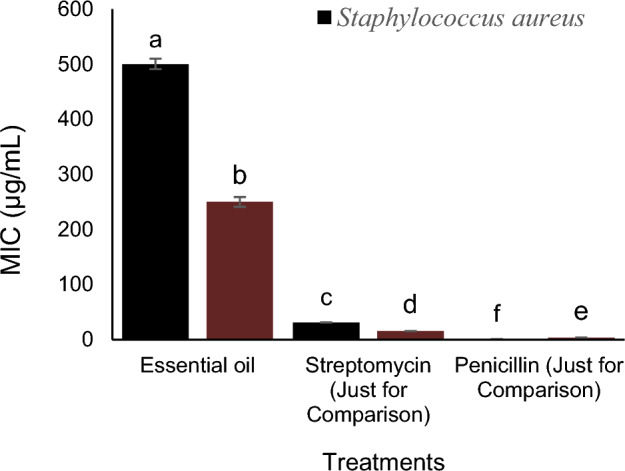
The MIC of Essential oil, Streptomycin (Just for Comparison), Penicillin (Just for Comparison) on *S. aureus* and *B. subtilis.*

**Figure 7:**
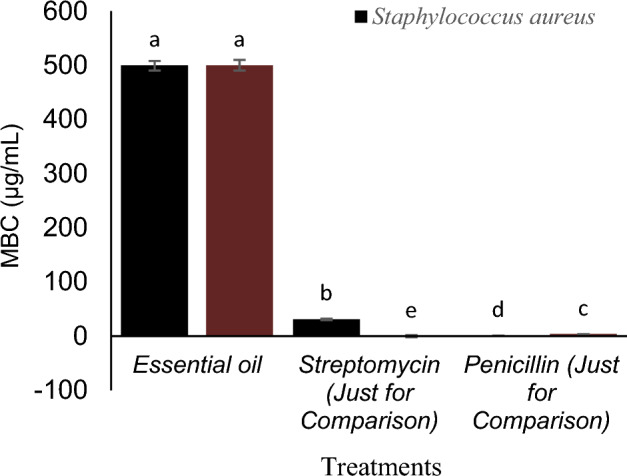
The MBC of Essential oil, Streptomycin (Just for Comparison), Penicillin (Just for Comparison) on *S. aureus* and *B. subtilis.*

The MIC and MBC values of EO were shown against *S. aureus* and *B. subtilis* (Figs. 6 and 7). The inhibitory activity of essential oil on *S. aureus* and *B. subtilis* is much lower than that of streptomycin and penicillin. Due to the MBC/MIC ratio (less than 4 for both bacteria), the antibacterial activity of this essential oil against the two studied bacteria is defined as bactericidal.

### Anti-fungal activity of essential oil

Descriptive statistics related to MIC and MFC of essential oil, nystatin and fluconazole on *A*. *flavus* are presented in Table 6. Based on the standard deviation values, the MIC was lower.

**Table 6:** Descriptive statistics related to The MIC of Essential oil, nystatin and fluconazole on *A. flavus* and *A. parasiticus.*

	Mean	Standard variation	Minimum	Maximum
MIC (µg/mL)	46.87	13.14	15.625	62.5
MBC (µg/mL)	54.68	16.58	7.8	125

Descriptive statistics related to MIC and MFC of essential oil, nystatin and fluconazole on and *A. parasiticus* are presented in Table 7. Based on the standard deviation values, the MIC was lower.

**Table 7:** Descriptive statistics related to The MFC of Essential oil, nystatin and fluconazole on *A. flavus* and *A. parasiticus.*

	Mean	Standard variation	Minimum	Maximum
MIC (µg/mL)	78.12	16.29	31.25	125
MFC (µg/mL)	109.37	23.14	15.625	250

**Figure 8:**
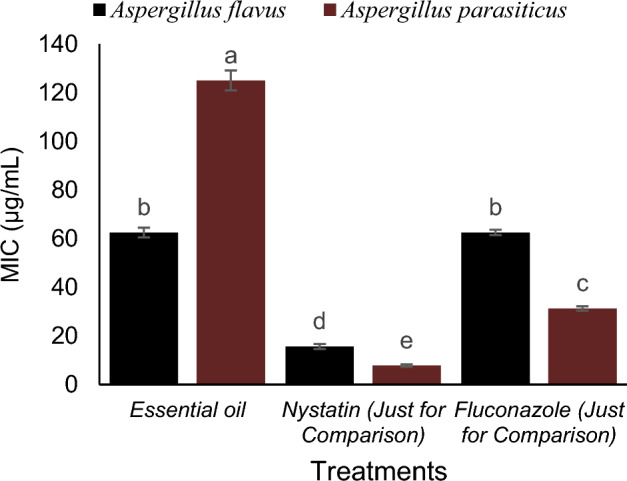
The MFC of Essential oil, nystatin and fluconazole on *A. flavus* and *A. parasiticus.*

**Figure 9:**
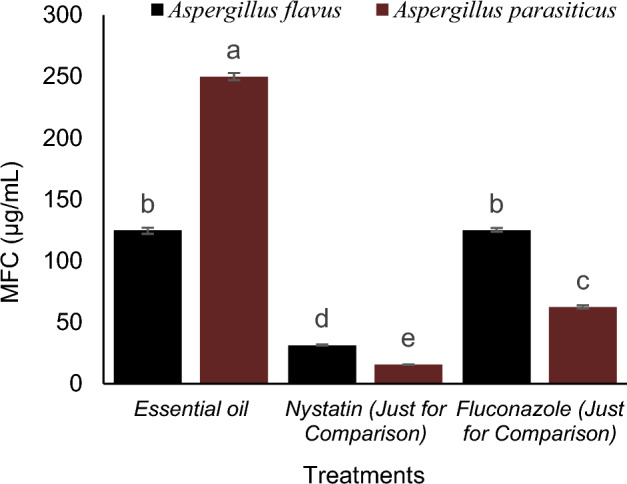
The MFC of Essential oil, nystatin and fluconazole on *A. flavus* and *A. parasiticus.*

The results showed that pistachio hull essential oil has an effective inhibitory impact against *A. flavus*, comparable to fluconazole. The results also indicate that the studied essential oil has inhibitory effects against *Aspergillus parasiticus*, which is much less inhibitory than the two antibiotics studied. Due to the MFC/MIC ratio (less than 4 for both fungi), the antifungal activity of this essential oil against the two fungi is defined as a fungicide (Figs. 8 and 9).

## Discussion

The pistachio hull EO can be broadly divided into five major chemical classes: (1) monoterpene hydrocarbons, (2) oxygenated monoterpenes, (3) sesquiterpenes (4) phenols, and (5) aliphatic hydrocarbons^10,32^.

According to a study, the significant constituents of pistachio green EO were α-pinene, α-terpinolene, limonene, and δ-3-carn (80%), with lesser levels of other volatile constituents such volatile compounds, such as α- phellandrene and borneyl acetate. α-Pinene, a terpene class natural chemical, can be found in pistachio hull EO in amounts up to 54^33^.

The chemical composition of pistachio green hull has been studied from different regions of Tunisia. This study reported that α-terpinolene is the main compound in the first region. In the second and third regions, α-pinene (42.5% and 43.8%, respectively) was the major compound^10^. For all samples, monoterpene hydrocarbons were predominant (more than 79.8% of the essential oil). In another study by this researcher, it was found that pistachio hull essential oil in all growth periods mainly contained monoterpene hydrocarbons, including α-pinene (15.0–47.4%) and terpinolene (32.2–51.1%)^34^. The hull essential oil of two pistachio samples from Shiraz city was analyzed^14^ and the results showed that the main components of EO from sample 1 were limonene (25.9%) and terpinolene (24.13%). Similarly, the main components of essential oil from sample 2 were limonene (47.69%) and terpinolene (24.08%). In another study, β-Caryophyllene, Myrcene, α-Pinene, Limonene and α-Humulene were respectively recognized as major constituents of the EO of *Pistacia khinjuk*^35^. The major constituents identified in the pistachio hull essential oil are α-pinene, α-terpinolene, limonene, and δ-3-carene (~ 80%), which are followed by smaller amounts of other volatile compounds, such as α-phellandrene, bornyl acetate, 2-carene, α-terpinene, α-terpineol, 2-furanmethanol, camphene, and terpinen-4-ol. The α-pinene is a natural compound of the terpene class that occurs in the essential oil of PGH extract at concentrations of up to 54%^33^.

The hull of pistachios is a well-known source of biologically active substances. It contains beneficial phytochemicals such as anthocyanins, flavan-3-ols, proanthocyanidins, flavonols, isoflavones, flavonoids, acetylene, and TPC^36,37^. These compounds possess biological antioxidants^34^ and antimicrobial activities^38–40^. In recent years, the natural phenolic and antioxidant compounds found in pistachio hull have attracted the interest of researchers. The purified compound (F13b1/PV-EA) has demonstrated cytotoxic effects against MCF-7 cells. This discovery could potentially influence the future of cancer treatment by offering a promising approach for the development of a combined anticancer drug treatment that could enhance induced apoptosis^41^. Mastic gum, derived from the stem and leaves of Pistacia lentiscus trees, is a resinous exudation that has been described as a combination of potent anticancer drugs. It aids in enhancing the anticancer properties of mastic gum and its primary components, while also emphasizing the diverse molecular mechanisms by which triterpenoids exert their remarkable anti-cancer effects^42^.

Very few investigations have been conducted on the TPC and anti-radical activity of pistachio shell EO. According to previous research^7^, the amount of TPC in the pistachio green hull was 205.68 mg of gallic acid per 100 g of the sample's dry weight, which is less than the obtained amount in the present study. Pistachio hull, especially the Ahmad-Aghaei cultivar, contains many phenolic compounds that can protect against oxidizing agents and pathogens^43^.

It has been reported that the concentration of EOs and extracts directly correlates with the amount of TPCs. TPCs significantly affect the percentage of free radical scavenging. Phenolic acids, such as gallic acid, exhibit their antioxidant properties in two ways: (1) donating a hydrogen atom, and (2) acting as an electron donor^28,44^. Pistachio hull, in general, is a significant source of natural antioxidants (quercetin, gallic acid, galloyl derivatives, theogallrides, α-pinene, pyrogallol, and α-terpinolene) with potentially beneficial antioxidant properties^45–47^. It has also been reported that compounds of cyanidin-3-O-galactoside, eriodectiol-7-O-glucoside, and epicatechin are present in pistachio hull seem to be the cause of its antioxidant capacity^29^. The most prevalent phenols in the green pistachio hull were catechins, gallic acid, rutin, and eriodictyol-7-O-glucoside^34,48^. The flavan-3-ols (catechins) and flavonols are in large amounts in the pistachio hull^45^. According to studies, phenolic acids, like gallic acid, demonstrate their antioxidant characteristics by functioning as electron and hydrogen atom donors^33,47^.

In a study, the antioxidant activity of pistachio hull extract and related polyphenols (phloroglucinol and gallic acid) were compared to the synthetic antioxidant (Trolox). The results showed that crude pistachio hull extract had lower antioxidant activity than individual polyphenols, but was similar to that of Trolox; thus, pistachio hull showed strong antioxidant activity^46^. Rajaei et al.^49^, have reported significantly higher antioxidant activity of purified pistachio hull extract (84.5% at 4 µg/mL) compared to crude extract (76.5% at 4 µg/mL). Also, the results confirmed the high antioxidant potential of pistachio hull extracts when compared to synthetic antioxidants (BHT 55.9% at 5 µg/mL and TBHQ 70.7% at 5 µg/mL).

Pistachios have been shown to contain 56 components, which account for 99.5% of the oil's overall makeup. Staphylococcus aureus was the most sensitive strain (MIC and MBC = 16 μg/ml) in the antibacterial findings. The relative antioxidant IC50 values were 19.03 ± 0.001 and 49.22 ± 0.005 μg/mL. For the MCF-7, PC3, and DU-145 cell lines, the IC50 values of the cytotoxic tests were 29.6, 37.3, and 41.1 μg/mL, respectively^35^. The IC_50_ of essential oil was 206.32 µl/L. In comparison to previous research^7^, the rate obtained in the current study was significantly lower. They reported the amount of pistachio hull EO at 3500 ppm. The observed difference can be due to differences in cultivar, soil conditions, climate, and degree of fruit ripening.

Azadedel et al.^50^ have reported the antioxidant properties of pistachio hull extracts of two cultivars, Kallehghuchi and Ohadi, with the addition of four solvents (acetone, methanol, ethanol, and water) with two methods (ultrasound and soaking). In their study, all pistachio hull extracts showed powerful antioxidant properties, so in the DPPH method, the antioxidant properties of the extracts were much higher than the gallic acid and ascorbic acid.

Due to the MBC/MIC ratio (less than 4 for both bacteria), the antibacterial activity of this essential oil against the two studied bacteria is defined as bactericidal. In a study, it was observed that phenolic compounds in the methanolic extract of pistachio green hull have an antimicrobial effect on *Bacillus cereus* and *S. aureus*^49^*.* The antimicrobial properties of the Kaleghoochi cultivar at the highest concentration (1200 μg/disc) were higher than the tetracycline^49^.

It has been demonstrated that phenolic chemicals found in the aqueous extract of pistachio hull have an inhibitory effect on a number of food-borne bacteria. In addition, the phenolics, flavonoid, and anthocyanin compounds were strongly associated with antibacterial activity^51^. Pistachio hull contain phenolic compounds that are rich in hydroxybenzoic acids (vanillic and gallic acids), flavonoids (catechins, quercetin, and apigenin), and tannins^37^. Gallic acid, the primary phenolic acid in pistachio hull extract, has antibacterial properties that are brought on by altering intracellular pH as a result of altered ion movement and by obstructing energy synthesis by interfering with the energy production system^52^. While apigenin inhibits DNA gyrase and protein dehydratase activity, quercetin alters membrane potential and enhances membrane permeability^53,54^. Additionally, tannins may attach to enzymes and damage cell membranes and metabolic pathways by doing so^55^.

One of the main components identified in pistachio hull essential oil is α-pinene, a strong antibacterial compound inhibiting bacteria that cause food-borne illness^56^. In research on six pathogenic strains (*B. cereus*, *Micrococcus luteus*, *S. aureus*, *E. coli*, *Pseudomonas aeruginosa*, and *Salmonella typhi*), the MIC of wild pistachio (*P*. *khinjuk*) hull EO was measured. It was found that *S. aureus* was the most sensitive strain^35^. In another work, the growth of *S. aureus* and *E*. *coli* was observed to be inhibited by pistachio green hull EO at a dose of 7.11 mg/ml^32^.

The antibacterial properties of different pistachio hull extracts have been reported on *S. aureus*^45^. The acetone extract exhibited stronger properties compared to other solvents. It showed more antimicrobial properties in lower dilutions. Thus, the MIC and MBC levels were 88.55 and 354.16 µg/mL, respectively^57^. The antimicrobial properties of green hull of walnuts showed antimicrobial properties against Gram-positive bacteria such as *S. aureus* and *B. cereus*.

The antifungal effect of different components of pistachio (leaves, stems, branches, bark, and kernel) extracts on other Candida species and reported that all extracts at a concentration of 16 μg/ml had a considerable antifungal effect against *C. albicans* and *C. parapsilosis* that it was close to the effective concentration of antibiotic control agents.

It has evaluated^58,59^ the antimicrobial activity of pistachio hull essential oil against different species of candida and compared it with various antifungal agents. According to their findings, the main active ingredients in pistachio hull essential oil were 3-Carene and D-limonene compounds, which demonstrated fungicidal activities in concentrations between 2.50 and 0.5 mg/ml.

Mahoney et al.^60^ found that hull is rich in phenolic compounds. These compounds, such as gallic acid, chlorogenic acid, caffeic acid, and tannic acid, can inhibit the growth of *A. flavus* and the production of aflatoxin B1.

The green hull of pistachio has also been reported to contain galatonin. Galatonins are hydrolyzable tannins mainly consisting of glucose esters (or other polyols) and gallic acid. Green peel tannin strongly inhibits the growth of *A. flavus*^61^. Also, among the compounds in pistachio hull essential oil, α-pinene has biological effect against bacteria, fungi, and insects. Myrcene has shown antifungal and insecticide effects. Limonene acts against fungi and insects^10^. Gallic acid and quercetin, two of the major phenolic compounds found in pistachio hull, induce the increase in permeability (or fluidity) of the outer and inner bacterial membrane, and disturbance of the membrane potential^62^.

The results of another research showed that phenolic and flavonoid compounds as secondary antioxidant metabolites in the green hull of pistachio cultivars play a key role in inhibiting *A. flavus* growth^61^. Fattahifar et al.^63^ reported increase in the count of mesophilic bacteria in the control and pistachio hull extract treated mushrooms increased respectively up to 5.93 and 5.73 log cfu/g during storage, but no growth of psychrophilic bacteria was observed during storage. Al-Juhaimi^64^ showed that the total aerobic microbial count of chicken burger was significantly reduced by increasing the concentration of the pistachio hull water extract. Furthermore, antimicrobial activity of lyophilized pistachio water extract against lactic acid bacteria, *S. aureus*, total viable microorganism, and fungi was observed.

## Conclusion

The findings of this study demonstrated the potential of pistachio hull EO as a cheap and available source of bioactive compounds. the result of this research were shown that The essential oil obtained from the pistachio hull can show good antioxidant and microbial properties but In order to confirm the results of this research and the use of pistachio shell essential oil, additional tests and clinical tests should be performed.

## Data Availability

All the data are embedded in the manuscript.

## References

[1] Fazeli-Nasab, B. & Sayyed, R. Z. in *Plant Growth Promoting Rhizobacteria for Sustainable Stress Management: Volume 1: Rhizobacteria in Abiotic Stress Management* (eds Sayyed, R. Z., Arora, N. K. & Reddy, M. S.) 21–34 (Springer, 2019).

[2] Karabulut F, Aydın S, Parray JA (2021). Interactions of antioxidant defense mechanisms developed by plants and microorganisms against pesticides. Micro Environer.

[3] Karabulut F, Parray JA, Mir MY (2021). Emerging trends for Harnessing plant metabolome and microbiome for sustainable food Production. Micro Environer.

[4] Alavi M, Hamblin MR, Aghaie E, Mousavi SAR, Hajimolaali M (2023). Antibacterial and antioxidant activity of catechin, gallic acid, and epigallocatechin-3-gallate: Focus on nanoformulations. Cell. Mol. Biomed. Rep..

[5] Aziziaram Z, Bilal I, Zhong Y, Mahmod AK, Roshandel MR (2021). Protective effects of curcumin against naproxen-induced mitochondrial dysfunction in rat kidney tissue. Cell. Mol. Biomed. Rep..

[6] Salehi-Sardoei A, Khalili H (2022). Nitric oxide signaling pathway in medicinal plants. Cell. Mol. Biomed. Rep..

[7] Mohammadi M, Ghorbani M, Beigbabaei A, Yeganehzad S, Sadeghi-Mahoonak A (2019). Investigation effects of extracted compounds from shell and cluster of pistachio nut on the inactivation of free radicals. Heliyon.

[8] Anonymous. *Agricultural Statistics *(*It was reported by Iranian Ministry of Agriculture*). (Tehran, Islamic Republic of Iran, 2016).

[9] Bohluli A, Nasserian AA, Valizadeh R, Eftekhar-Shahrodi F (2010). The chemical composition, in vitro gas production and digestibility of pistachio by-products. Iran. J. Anim. Sci. Res..

[10] Chahed T (2007). Comparison of pistachio hull essential oils from different Tunisian localities. Ital. J. Biochem..

[11] Mohammadi-Moghaddam T, Razavi SM, Malekzadegan F, Ardekani AS (2009). Chemical composition and rheological characterization of *pistachio* green hull's marmalade. J. Texture Stud..

[12] Hepsag F, Golge O, Kabak B (2014). Quantitation of aflatoxins in pistachios and groundnuts using HPLC-FLD method. Food Control.

[13] Goli AH, Barzegar M, Sahari MA (2005). Antioxidant activity and total phenolic compounds of pistachio (*Pistachia vera*) hull extracts. Food Chem..

[14] Ayatollahi SZ, Yousefi G, Badr P (2021). Essential oil analysis of *Pistacia vera* L. hull samples from Iran. Trends Pharm. Sci..

[15] Moradi MG, Hokmabadi H, Mirabou Alfathi M (2010). Density fluctuations of two major *Aspergillus* species airborne spores in pistachio growing regions of Iran. Int. J. Nuts Related Sci..

[16] Marín S, Ramos AJ, Sanchis V (2012). Modelling *Aspergillus flavus* growth and aflatoxins production in pistachio nuts. Food Microbiol..

[17] Shakerardekani A, Karim R, Mirdamadiha F (2012). The effect of sorting on aflatoxin reduction of pistachio nuts. J. Food Agric. Environ..

[18] Habibi A (2021). *Aspergillus* species in retail samples of pistachio, walnut, and hazelnut in Kerman. Iran. Mycologia Iranica.

[19] Panahi B, Khezri M (2011). Effect of harvesting time on nut quality of pistachio (*Pistacia vera* L.) cultivars. Sci. Hortic..

[20] Baazeem A, Garcia-Cela E, Medina A, Magan N (2021). Interacting abiotic factors affect growth and aflatoxin b1 production profiles of *Aspergillus flavus* strains on pistachio-based matrices and pistachio nuts. Front. Microbiol..

[21] Bensassi F, Rhouma A, Ghrab M, Bacha H, Rabeh Hajlaoui M (2010). Evaluation of cultivar susceptibility and storage periods towards aflatoxin B1 contamination on pistachio nuts. Mycotoxin Res..

[22] Amaike S, Keller NP (2011). *Aspergillus flavus*. Annu. Rev. Phytopathol..

[23] Chahed T (2008). Composition of Tunisian pistachio hull essential oil during fruit formation and ripening. J. Essent. Oil Res..

[24] Heidary-Sharifabad A, Zarchi MS, Zarei G (2021). ICPTC: Iranian commercial pistachio tree cultivars standard dataset. Data Brief.

[25] Araújo FM (2017). Antibacterial activity and chemical composition of the essential oil of *Croton heliotropiifolius* Kunth from Amargosa, Bahia Brazil. Ind. Crops Prod..

[26] Bajalan I, Zand M, Goodarzi M, Darabi M (2017). Antioxidant activity and total phenolic and flavonoid content of the extract and chemical composition of the essential oil of *Eremostachys laciniata* collected from Zagros. Asian Pac. J. Trop. Biomed..

[27] Luís Â, Duarte A, Gominho J, Domingues F, Duarte AP (2016). Chemical composition, antioxidant, antibacterial and anti-quorum sensing activities of *Eucalyptus globulus* and *Eucalyptus radiata* essential oils. Ind. Crops Prod..

[28] Shahdadi F, Payandeh M, Salehi Sardoei A (2021). Comparison of antioxidant activity of *Dracocephalum polychaetum* Bornm and *Nepeta cataria* L. and their effect on probiotic bacteria in a simulated gastrointestinal environment. J. Med. Microbiol. Infec. Dis..

[29] Tomaino A (2010). Antioxidant activity and phenolic profile of pistachio (*Pistacia vera* L., variety Bronte) seeds and skins. Biochimie.

[30] Sefidgar SAA (2015). Evaluation of antimicrobial activity of alcoholic and aqueous extracts from common hop (*Humulus lupulus*) and oak (*Quercus castaneifolia*). J. Arak Univ. Med. Sci..

[31] Gharanjic I (2015). Antifungal activity of Kiwi alcoholic extract on saprophytes and dermatophytes fungi. J. Rafsanjan Univ. Med. Sci..

[32] Smeriglio A (2017). In vitro evaluation of the antioxidant, cytoprotective, and antimicrobial properties of essential oil from *Pistacia vera* L. Variety Bronte Hull. Int. J. Mol. Sci..

[33] Küsmenoglu S, Baser K, Özek T (1995). Constituents of the essential oil from the hulls of *Pistacia vera* L. J. Essent. Oil Res..

[34] Maged MA, Kishk Y, Khalil H, Gibriel A (2007). Determination of total phenolics, flavonoids and free radical scavenging activities of pistachio and tomato by-products. AOAS.

[35] Taghizadeh SF (2018). Chemical composition, antibacterial, antioxidant and cytotoxic evaluation of the essential oil from pistachio (*Pistacia khinjuk*) hull. Microb. Pathog..

[36] Kilic IH (2016). A significant by-product of the industrial processing of pistachios: Shell skin-RP-HPLC analysis, and antioxidant and enzyme inhibitory activities of the methanol extracts of *Pistacia vera* L. shell skins cultivated in Gaziantep. Turkey. RSC Adv..

[37] Arjeh E, Akhavan H-R, Barzegar M, Carbonell-Barrachina ÁA (2020). Bio-active compounds and functional properties of pistachio hull: A review. Trends Food Sci. Technol..

[38] Fazeli-Nasab B, Shahraki-Mojahed L, Hassanzadeh MA, Bidarnamani F (2022). Investigation of antimicrobial activity of medicinal plant extracts on *Bacillus cereus* isolated from soil. Gene, Cell Tissue.

[39] Wang Y (2022). Chemical constituents and pharmacological activities of medicinal plants from Rosa genus. Chin. Herb. Med..

[40] Chhetri G, Kim I, Kim J, So Y, Seo T (2022). Chryseobacterium tagetis sp. nov., a plant growth promoting bacterium with an antimicrobial activity isolated from the roots of medicinal plant (*Tagetes patula*). J. Antibiot..

[41] Seifaddinipour M, Farghadani R, Namvar F, Bin Mohamad J, Muhamad NA (2020). In vitro and in vivo anticancer activity of the most cytotoxic fraction of pistachio hull extract in breast cancer. Molecules.

[42] Seifaddinipour M, Farghadani R, Namvar F, Mohamad J, Abdul Kadir H (2018). Cytotoxic effects and anti-angiogenesis potential of pistachio (*Pistacia vera* L.) hulls against MCF-7 human breast cancer cells. Molecules.

[43] Nadernejad N, Ahmadimoghadam A, Hosseinifard J, Pourseyedi S (2012). Phenylalanin ammonia-lyase activity, total phenolic and flavonoid content in flowers, leaves, hulls and kernels of three pistachio (*Pistacia vera* L.) cultivars. Am. Eurasian J. Agric. Environ. Sci..

[44] Ghorbani A, Esmaeilizadeh M (2017). Pharmacological properties of *Salvia officinalis* and its components. J. Tradit. Complement. Med..

[45] Barreca D (2016). Evaluation of the nutraceutical, antioxidant and cytoprotective properties of ripe pistachio (*Pistacia vera* L., variety Bronte) hulls. Food Chem..

[46] Lalegani S, Gavlighi HA, Azizi MH, Sarteshnizi RA (2018). Inhibitory activity of phenolic-rich pistachio green hull extract-enriched pasta on key type 2 diabetes relevant enzymes and glycemic index. Food Res. Int..

[47] Seifzadeh N (2019). Evaluation of polyphenolic compounds in membrane concentrated pistachio hull extract. Food Chem..

[48] Aminger W (2021). Preservice secondary science teachers’ implementation of an NGSS practice: Using mathematics and computational thinking. J. Sci. Teach..

[49] Rajaei A, Barzegar M, Mobarez AM, Sahari MA, Esfahani ZH (2010). Antioxidant, anti-microbial and antimutagenicity activities of pistachio (*Pistachia vera*) green hull extract. Food Chem. Toxicol..

[50] Azadedel S, Hanachi P, Saboora A (2018). Investigation on antioxidant activity of pistachio (*Pistacia vera* L.) skin extraction. J. Plant Res. (Iran. J. Biol.).

[51] Elhadef K (2021). Tunisian pistachio hull extracts: Phytochemical content, antioxidant activity, and foodborne pathogen inhibition. J. Food Qual..

[52] Bouarab-Chibane L (2019). Antibacterial properties of polyphenols: Characterization and QSAR (quantitative structure–activity relationship) models. Front. Microbiol..

[53] Dzik S (2020). COVID-19 convalescent plasma: Now is the time for better science. Transfus. Med. Rev..

[54] Fazeli-Nasab B (2021). Biological evaluation of coronaviruses and the study of molecular docking, linalool, and thymol as orf1ab protein inhibitors and the role of SARS-CoV-2 virus in bioterrorism. J. Ilam Univ. Med. Sci..

[55] Rempe CS, Burris KP, Lenaghan SC, Stewart CN (2017). The potential of systems biology to discover antibacterial mechanisms of plant phenolics. Front. Microbiol..

[56] Dai J, Zhu L, Yang L, Qiu J (2013). Chemical composition, antioxidant and antimicrobial activities of essential oil from *Wedelia prostrata*. EXCLI J..

[57] Fernández-Agulló A (2013). Influence of solvent on the antioxidant and antimicrobial properties of walnut (*Juglans regia* L.) green husk extracts. Ind. Crops Prod..

[58] Mohammadi Moghaddam M, Rezaee S, Mohammadi AH, Zamanizadeh HR, Moradi M (2020). Relationship between *Aspergillus flavus* growth and aflatoxin B1 and B2 production with phenolic and flavonoid compounds in green hull and kernels of pistachio cultivars. Appl. Entomol. Phytopathol..

[59] D’Arrigo M (2019). In vitro evaluation of the activity of an essential oil from *Pistacia vera* L. variety Bronte hull against Candida sp. BMC Complement Altern. Med..

[60] Mahoney, N., Molyneux, R. & Campbell, B. in *Proceeding of the 2nd Fungal Genomic, 3rd Fumonisin Elimination and 15th Aflatoxin Elimination Workshop.*

[61] Latha P, Sudhakar P, Sreenivasulu Y, Naidu PH, Reddy PV (2007). Relationship between total phenols and aflatoxin production of peanut genotypes under end-of-season drought conditions. Acta Physiologiae Plantarum.

[62] Simoes M, Bennett RN, Rosa EA (2009). Understanding antimicrobial activities of phytochemicals against multidrug resistant bacteria and biofilms. Nat. Prod. Rep..

[63] Fattahifar E, Barzegar M, Gavlighi HA, Sahari M (2018). Evaluation of the inhibitory effect of pistachio (*Pistacia vera* L.) green hull aqueous extract on mushroom tyrosinase activity and its application as a button mushroom postharvest anti-browning agent. Postharvest Biol. Technol..

[64] Al-Juhaimi F (2017). Effect of pistachio seed hull extracts on quality attributes of chicken burger. CyTA-J. Food.

